# Supplementary material for: Optical Biomedical Imaging Reveals Criteria for Violated Liver Regenerative Potential
Source: Cells. 2023 Feb 2;12(3):479. doi: 10.3390/cells12030479 (PMC9914457; doi:10.3390/cells12030479)
Supplement: Supplementary file 1 [file cells-12-00479-s001.zip › cells-2099860-supplementary.pdf]

Supplementary Materials

Optical biomedical imaging reveals criteria for violated liver regenerative potential

Svetlana Rodimova[1,2*], Nikolai Bobrov [1,3], Artem Mozherov[1,2], Vadim Elagin[1], Maria Karabut[1], Ilya Shchechkin[1,2], Dmitry Kozlov[1,2], Dmitry Krylov[1,2], Alena Gavrina[1,2], Vladimir Zagainov[1,4], Elena Zagaynova[1,2], Daria Kuznetsova[1,2]

1 Institute of Experimental Oncology and Biomedical Technologies, Privolzhsky Research Medical University, 10/1 Minin and Pozharsky Sq., Nizhny Novgorod 603000, Russia
2 N.I. Lobachevsky Nizhny Novgorod National Research State University, 23 Gagarina Ave., Nizhny Novgorod 603022, Russia
3 The Volga District Medical Centre of Federal Medical and Biological Agency, 14 Ilinskaya St., Nizhny Novgorod 603000, Russia
4 Nizhny Novgorod Regional Clinical Oncologic Dispensary, Delovaya St., 11/1, Nizhny Novgorod 603126, Russia
* Correspondence: srodimova123@gmail.com

The goodness of fit model assessment

Goodness of fit model was evaluated on the basis of $\chi 2$ values, which were in the range of 0.9 - 1.2. Below we have provided new Figures showing the typical tri-exponential fitting and fluorescence decay of NAD(P)H.

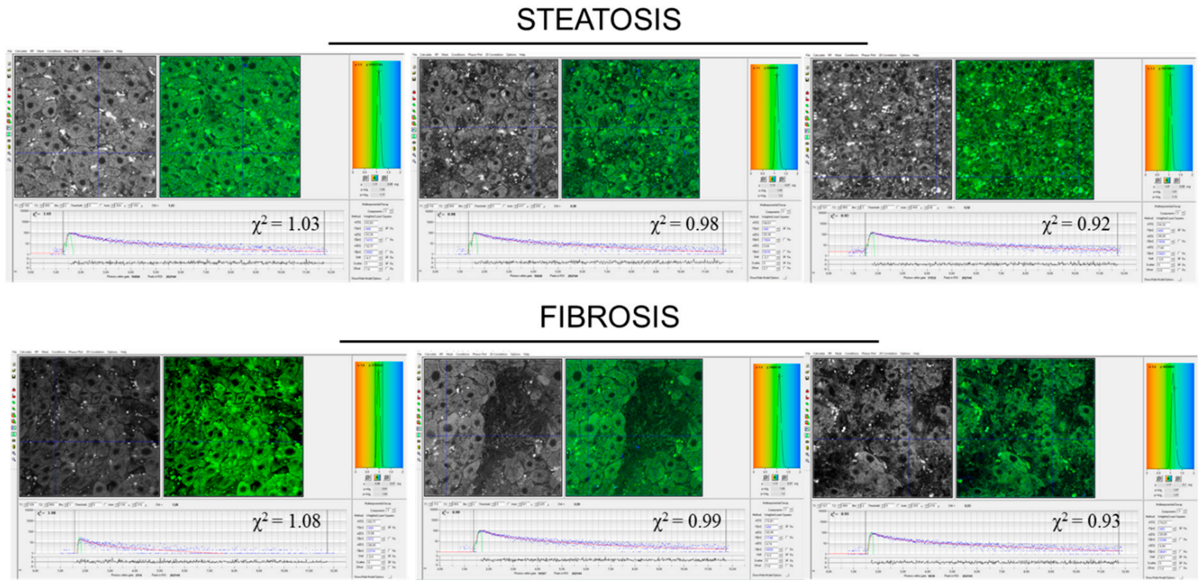

Figure S1. Image of typical tri-exponential fitting and fluorescence decay of NAD(P)H. The images were obtained using SPCImage software. The values of the goodness of the fit have been enlarged and placed on the right side of each image.

Biochemical blood tests

In the case of steatosis, biochemical blood tests showed that the ALP level, which is associated with liver tissue damage, exceeded its normal range at every monitoring time point, except for the 7th day after PH in the 12th week. The AST increased on the 3rd day of every week of the pathology. The ALT significantly increased on the 3rd day after PH during the 2nd and 6th weeks, and also increased in the 9th week, before PH. The parameters of urea and creatinine reflect the detoxification function of the liver. The total protein decreased at almost every stage of both the pathology and regeneration, indicating a decrease in the synthetic functions of the pathological and regenerating liver. Most biochemical parameters reflecting lipid metabolism did not significantly exceed their normal range. The exceptions to this were an elevated HDL value in the 3rd week before resection, and an elevated LDL on the 3rd day after PH during the 9th and 12th weeks. TG increased on the 3rd day after PH during the 6th week, and by the 9th before PH. These results were not consistent with the data from the histological analysis and multiphoton microscopy, where we had observed a significant increase of lipid infiltration in the hepatocytes. The results of a biochemical blood analysis at different stages of steatosis with induced regeneration are shown in Figure S1.

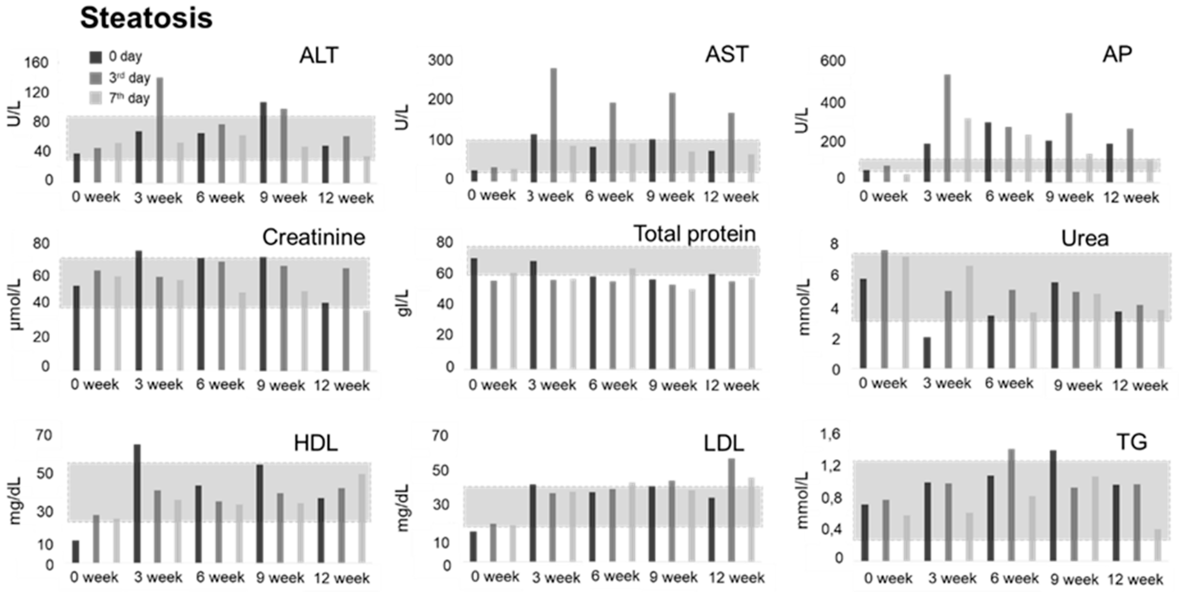

Figure S2. Biochemical parameters in the blood serum of rats at different stages of steatosis with induced regeneration. The area marked with a dotted line reflects the range of physiological values for each biochemical parameter under study.

Biochemical blood tests for hepatic fibrosis showed that the ALP, significantly exceeded the normal range at each stage of the pathology and regeneration except for day 0 and the 7th day after PH during the 6th week of fibrosis. The AST parameter also showed high values, predominantly on the 3rd day after PH, which can be explained by postoperative injuries. In addition, the AST parameter was significantly elevated during the 6th week, before PH. The ALT parameter did not significantly exceed its normal range, with the exception of the 6th week, before PH.

The values of the urea and creatinine parameters did not significantly go beyond the range of their normal values, with the exception of a sharp jump in creatinine on the 3rd day after PH during the 2nd week, and a slight excess over the normal values for urea during the 6th week, before PH. However, the total protein was reduced on the 3rd and 7th days after PH at each stage of the induced pathology, indicating disturbed synthetic function of the regenerating liver.

The biochemical blood parameters reflecting lipid metabolism did not significantly exceed their normal ranges, however, there was a slight increase in LDL during the 4th week, before PH and an increase in HDL in the 6th week before PH. Again, such results are not consistent with the data of from the histological analysis and multiphoton microscopy, where we had observed significant accumulation of lipid droplets in the hepatocytes at every stage of the pathology. The results of the biochemical blood tests at different stages of fibrosis with induced regeneration are shown in Figure S2.

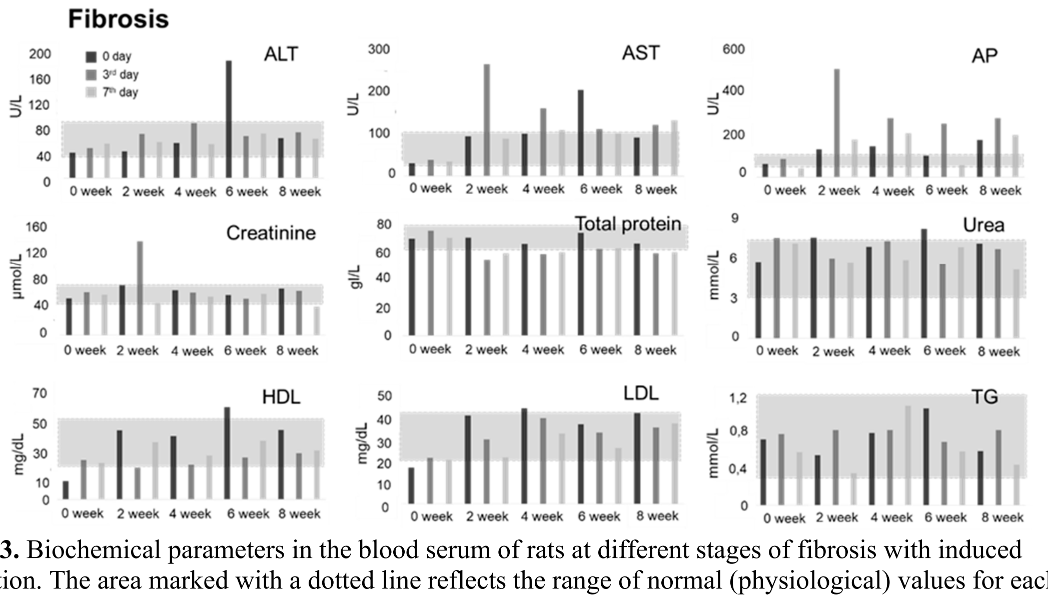

Figure S3. Biochemical parameters in the blood serum of rats at different stages of fibrosis with induced regeneration. The area marked with a dotted line reflects the range of normal (physiological) values for each biochemical parameter under study.

Table S1. Fluorescence lifetime values of the bound form of NADH (t2) and NADPH (t3) in hepatocytes

STEATOSIS		t2, ps	t3, ps	tm, ps
Control	0 day	2276 ± 101	4105 ± 317	1276 ± 39
	3rd day	2194 ±113	4130 ± 421	1387 ± 43
	7th day	2094 ± 96	3861 ± 276	1312 ± 55
3rd week	0 day	1968 ± 108	3931 ± 287	973 ± 40
	3rd day	2031 ± 134	3660 ± 257	1167 ± 51
	7th day	1997 ± 115	3761 ± 304	1130 ± 49
6th week	0 day	2054 ± 129	4071 ± 337	1068 ± 60
	3rd day	2022 ± 149	4156 ± 245	1079 ± 58
	7th day	2023 ± 111	4063 ± 482	1036 ± 45
9th week	0 day	1992 ± 115	4220 ± 368	1079 ± 59
	3rd day	1999 ± 120	4065 ± 305	1129 ± 55
	7th day	1992 ± 95	4202 ± 245	1078 ± 86
12th week	0 day	1987 ± 132	4220 ± 582	1055 ± 46
	3rd day	2038 ± 100	4068 ± 290	1163 ± 56
	7th day	2004 ± 127	4494 ± 687	1099 ± 44

FIBROSIS		t2, ps	t3, ps	tm, ps
Control	0 day	2276 ± 101	4105 ± 317	1276 ± 39
	3rd day	2194 ±113	4130 ± 421	1387 ± 43
	7th day	2094 ± 96	3861 ± 276	1312 ± 55
2nd week	0 day	1928 ± 160	3742 ± 327	985 ± 78
	3rd day	1825 ± 220	3546 ± 328	1030 ± 70
	7th day	1723 ± 232	3705 ± 385	951 ± 61
4th week	0 day	1973 ± 149	3703 ± 311	948 ± 79
	3rd day	1886 ± 179	3445 ± 427	1027 ± 50
	7th day	2005 ± 193	3498 ± 406	1078 ± 67
6th week	0 day	1889 ± 141*	3868 ± 285	983 ± 45
	3rd day	1708 ± 200*	3491 ± 169	1126 ± 55
	7th day	1903 ± 183*	3929 ± 237	974 ± 55
8th week	0 day	1797 ± 207*	3564 ± 186	1029 ± 68
	3rd day	1702 ± 201*	3536 ± 227	1113 ± 65
	7th day	1656 ± 211*	3504 ± 203	1039 ± 46

* - statistically significant differences compared to the corresponding time point for normal regeneration (control), p-value ≤0.05.

Table S2. The intensity of the NAD(P)H autofluorescence in the liver tissue with hepatic steatosis at different stages of regeneration

STEATOSIS		High NAD(P)H autofluorescence intensity (I_1)	Low NAD(P)H autofluorescence intensity (I_2)	Ratio (I_1/I_2)
Normal regeneration	0 day	310.57±3.15	-	-
	3rd day	351.28±4.73	-	-
	7th day	320.84±3.41	-	-
3rd week	0 day	208.62±2.78*	57.55±2.16#	3.73±1.46
	3rd day	197.87±1.93*	49.25±7.59#	4.18±1.28
	7th day	150.12±1.68*	50.73±3.81#	3.20±1.76
6th week	0 day	196.14±4.66*	59.63±2.41#	3.90±1.48
	3rd day	178.94±4.05*	44.43±1.43#	4.24±1.71
	7th day	208.16±4.97*	62.37±2,24#	3.55±1.17
9th week	0 day	247.86±5.23*	64.51±2.08#	3.81±1.21
	3rd day	258.24±5.66*	56.41±2.48#	6.24±2.68
	7th day	328.59±4.98	93.94±2.71#	3.66±1.24
12th week	0 day	280.42±3.39*	76.60±3.32#	4.32±1.19
	3rd day	350.97±4.84	113.64±3.64#	3.39±1.54
	7th day	344.64±6.59	147.76±1.06#	3.33±2.04

* - statistically significant difference from the corresponding time point for a normal liver, # - statistically significant difference from the zone with a high intensity of the NAD(P)H autofluorescence, p ≤ 0.05.

Table S3. The intensity of the NAD(P)H autofluorescence in the liver tissue with fibrosis at different stages of regeneration

FIBROSIS		High NAD(P)H autofluorescence intensity (I_1)	Low NAD(P)H autofluorescence intensity (I_2)	Ratio (I_1/I_2)
Normal regeneration	0 day	310.57±3.15	-	-
	3rd day	351.28±4.73	-	-
	7th day	320.84±3.41	-	-
3rd week	0 day	225.53±6.54*	52.43±2.23#	4.66±2.00
	3rd day	197.02±5.51*	52.73±1.94#	3.60±1.11
	7th day	216.19±4.68*	71.72±1.64#	3.36±0.85
6th week	0 day	117.38±3.91*	31.69±1.49#	4.04±1.66
	3rd day	137.11±2.94*	36.11±1.09#	4.49±2.37
	7th day	73.29±1.87*	21.33±7.26#	4.93±2.37
9th week	0 day	192.52±4.36*	70.68±4.36#	3.54±2.05
	3rd day	177.65±5.56*	46.68±5.29#	3.66±2.21
	7th day	298.56±3.28*	87.74±2.83#	3.99±1.57
12th week	0 day	218.46±5.88*	52.80±2.69#	4.19±1,67
	3rd day	223.58±6.01*	92.96±2.62#	2.96±1,33
	7th day	283.69±3.24*	62.06±7.05#	4.07±1,31

[*] - statistically significant difference from the corresponding time point for a normal liver, [#] - statistically significant difference from the zone with a high intensity of the NAD(P)H autofluorescence, $p \leq 0.05$.
